# Adversity, social support and risk of self-harm during the COVID-19 pandemic

**DOI:** 10.1192/bjo.2022.553

**Published:** 2022-07-25

**Authors:** Rohan Borschmann, Paul A. Moran

**Affiliations:** Justice Health Unit, Centre for Health Equity, Melbourne School of Population and Global Health, University of Melbourne, Australia; and Centre for Adolescent Health, Murdoch Children's Research Institute, Melbourne, Australia; and Department of Psychiatry, University of Oxford, Warneford Hospital, Oxford, UK; and Melbourne School of Psychological Sciences, University of Melbourne, Australia; Centre for Academic Mental Health, Department of Population Health Sciences, Bristol Medical School, University of Bristol, UK

**Keywords:** Self-harm, social deprivation, COVID-19, adversity, loneliness

## Abstract

Little is known about the degree to which social factors interact with COVID-19-related adversity to increase the risk of self-harm thoughts and behaviours. Using data derived from a UK cohort study, Paul & Fancourt found that loneliness was associated with an increase in the odds of self-harm thoughts and behaviours, whereas high-quality social support protected against self-harm thoughts and behaviours. The authors concluded that it is the quality of social support and interactions, rather than the act of engaging in social interaction per se, that protects against self-harm in the context of adversity. The COVID-19 pandemic may exert longer-lasting effects on population mental health, and continued surveillance of mental health, including self-harm status, will be essential. If accompanied by appropriate measures of the availability and quality of social support, such monitoring could also inform the development of more effective adaptive interventions for those at risk of engaging in self-harm.

Self-harm is a significant global public health problem and, in many high-income countries, there has been a clear increase in its prevalence over the past decade.^1^ It is associated with numerous adverse health and social outcomes,^2^ including an increased risk of death by suicide^3^ and other causes.^4^ Self-harm is often precipitated by stress, and a growing body of work is documenting the association between adversity related to the COVID-19 pandemic and self-harm.^5,6^ Thus far, the evidence has been mixed, with some studies reporting an increase in self-harm since the onset of the pandemic^7^ and other studies reporting a decrease.^8^ This inconsistent messaging may be explained by: (a) the fact that most self-harm does not lead to help-seeking behaviour;^9^ (b) differing methods of case ascertainment across studies; and (c) the possibility that any reported decreases in self-harm since the onset of COVID-19 in 2020 may be associated with pandemic-related lockdowns/quarantines and wider changes in service utilisation, rather than a true decline in incidence.

## Paul & Fancourt's study of the interaction between social factors, adversity and risk of self-harm

Despite the growth in research examining mental health during the pandemic, little is known about the degree to which social factors interact with adversity to influence the risk of self-harm thoughts and behaviours. In this issue of *BJPsych Open*, Elise Paul and Daisy Fancourt^10^ report findings from the UCL COVID-19 Social Study to address this gap in knowledge. Using data from 49 227 adults in the UK, they examined how self-reported changes in four social factors (social support quality, loneliness, face-to-face social interaction for ≥15 min and telephone/video-based social interaction for ≥15 min) were associated with changes in self-harm thoughts and behaviours over time. They then examined how these four factors interacted with adversities (and worries about adversities) to increase the risk of self-harm thoughts and behaviours.

The authors found that increased loneliness was associated with a four-fold increase in the odds of self-harm thoughts and a doubling in the odds of self-harm behaviour. In contrast, better quality social support was associated with a 45% reduction in the odds of self-harm thoughts and a 29% reduction in the odds of self-harm behaviour. Face-to-face contact was associated with a very small increase in the likelihood of self-harm thoughts (raising intriguing questions about the nature of face-to-face contact and with whom this was had) and telephone/video contact was associated with a very small decrease in the likelihood of such thoughts. No association was observed between either face-to-face or telephone/video contact and the likelihood of engaging in self-harm behaviour. The authors concluded that it is the quality of social support and interactions – rather than the act of engaging in social interaction per se – that can protect against the likelihood of self-harm in the context of adversity.

As noted by the authors, key limitations included the use of a non-random sample (although findings were weighted to enhance representativeness), the likely under-ascertainment of self-harm due to the wording and timing of the self-harm questions, and the fact that the study did not capture the method(s) of self-harm in which respondents engaged.

## Anticipatory anxiety and loneliness

One finding of particular interest in Paul & Fancourt's study was that worrying about adversity was more strongly associated with self-harm than the reported experience of adversity. This suggests that respondents may have been experiencing anticipatory anxiety, a phenomenon that can occur when a person experiences increased anxiety and stress when thinking about an outcome that may (or may not) happen in the future. At a population level, mitigating such anticipatory anxiety is challenging because negative events typically gain disproportionate attention from news outlets.^11^ However, although research has demonstrated that people with more pessimistic views appear to confirm such views by selecting more negative news stories,^12^ these effects can be reduced by better informing people about the biases underlying news production (i.e. enhancing news media literacy in the general population). Ultimately, there may be a need for us all to be more exposed to more visible reminders of the sources of support and help that are available in the event of future adversity, such as (in the UK) the Samaritans and Citizens Advice.

The finding that loneliness exacerbated the impact of adversity on self-harm lends support to previous research examining the relationship between loneliness, self-harm and suicidal thoughts and behaviours. In 2020, a systematic review and meta-analysis of prospective studies concluded that loneliness was a significant predictor of both suicidal thoughts and behaviour,^13^ and research conducted since the onset of the pandemic has demonstrated that loneliness resulting from the pandemic has exerted a similar influence on self-harm.^14^

## Implications

The COVID-19 pandemic may lead to some longer-lasting effects on population mental health, and continued surveillance of mental health – including self-harm thoughts and behaviours – via repeated, population-based, data collection efforts will be essential. If accompanied by appropriate measures of the quality of social support, such monitoring could also inform the development and delivery of more effective adaptive interventions.

## Data Availability

Data availability is not applicable to this article as no new data were created or analysed in this study.
